# Supplementary material for: Revised Injury Severity Classification II (RISC II) is a predictor of mortality in REBOA-managed severe trauma patients
Source: PLoS One. 2021 Feb 10;16(2):e0246127. doi: 10.1371/journal.pone.0246127 (PMC7875379; doi:10.1371/journal.pone.0246127)
Supplement: S1 Appendix — (DOCX) [file pone.0246127.s001.docx]

Variables included in the model of the RISC II ^10^

| **Variable** | **Description** |
| --- | --- |
| Worst injury, second-worst injury | AIS injury severity level; if only one injury was coded, the second-worst injury was set to zero |
| Head injury | AIS injury severity level of the body region ‘head’ as defined for the ISS score |
| Age | Age in years at the time of accident, 10 categories |
| Sex | Males / females |
| ASA | Pre-trauma ASA (American Society of Anesthesiologists) score |
| Pupil reactivity | brisk, sluggish, and none. The first pre-hospital assessment was used; if missing, assessment on admission was used |
| Pupil size | normal, anisocoria, and bilateral dilated. The first pre-hospital assessment was used; if missing, assessment on admission was used |
| Motor function | normal (6 points in GCS); directed (4-5); non-directed (2-3), and none (1). The first pre-hospital assessment was used if available; if missing, assessment on admission was used in non-intubated cases |
| Mechanism | Blunt or penetrating mechanism of injury |
| Blood pressure | Systolic blood pressure (mmHg), first measurement after admission; in case of missing values, the first pre-hospital measurement was used |
| Coagulation: INR | International normalized ratio (INR); first measurement after admission |
| Acidosis: base deficit | Base deficit, or base excess (mEq/l); first measurement after admission |
| Blood: haemoglobin | Haemoglobin (g/dl); first measurement after admission |
| CPR | Cardiopulmonary resuscitation (CPR), performed pre-hospitally in case of cardiac arrest (not in the emergency room) |
